# The kinase receptor-interacting protein 1 is required for inflammasome activation induced by endoplasmic reticulum stress

**DOI:** 10.1038/s41419-018-0694-7

**Published:** 2018-05-29

**Authors:** Liang Tao, Hongfa Lin, Jingjing Wen, Qi Sun, Yan Gao, Xi Xu, Junsong Wang, Jianfa Zhang, Dan Weng

**Affiliations:** Center for Molecular Metabolism, Nanjing University of Science & Technology, 200 Xiaolingwei Street, Nanjing, 210094 China

## Abstract

Endoplasmic reticulum (ER) stress contributes to the development and progression of many chronic inflammatory diseases, including type 2 diabetes, obesity, atherosclerosis, neurodegenerative diseases, and cancer. ER stress has been reported to induce inflammasome activation and release of mature IL-1β, which contributes to many inflammatory diseases. The molecular mechanisms that activate the inflammasome during ER stress are still poorly understood. Here we report that the kinase receptor-interacting protein 1 (RIP1) plays an important role in ER stress-induced activation of inflammasome. Inhibition of RIP1 kinase activity by Necrostatin-1 or siRNA-mediated RIP1 knockdown significantly reduced ER stress-induced caspase-1 cleavage and IL-1β secretion in both bone marrow-derived macrophages (BMDMs) and J774A.1 macrophages. We speculate that the mitochondria fission factor dynamin-related protein 1 (DRP1) and reactive oxygen species (ROS) might function as the effectors downstream of RIP1 to mediate inflammasome activation. Our study reveals a critical role for RIP1 in regulating ER stress-induced inflammation responses, and proposes RIP1 as a potential pharmaceutical target to treat diseases resulting from unresolved ER stress-related inflammation.

## Introduction

The endoplasmic reticulum (ER), which functions as the main cellular endomembrane organelle for protein folding and lipid synthesis, has also been suggested to be a sensitive stress sensor in eukaryotic cells. Various internal or external perturbants, such as glucose deprivation, disruption of calcium homeostasis, metabolic disorder or microbial infection, can lead to accumulation of unfolded or misfolded proteins in the ER lumen, resulting in ER stress^1,2^. Under such stress conditions, a cellular pathway known as the unfolded protein response (UPR) is activated to resolve the stress and restore ER homeostasis. The UPR pathway includes three signaling branches mediated by three ER-localized receptors IRE1α, PERK, and ATF6, which aim to resolve the protein-folding defect of ER through modifying the cellular transcriptional and translational programs^3,4^.

In addition to UPR pathway, ER stress can also cause inflammatory responses. Accumulating evidence suggests that the interplay between ER stress and inflammation is involved in the development and progression of various diseases, including type 2 diabetes, obesity, arthritis, neurodegenerative diseases, and cancer^2,4,5^. Therefore, the clarification of the mechanisms of ER stress-induced inflammation will be helpful to identify promising therapeutic targets for these diseases. Several studies have reported that ER stress can induce the activation of inflammasome, leading to the maturation and release of the proinflammatory cytokine IL-1β^6–9^. However, the molecular mechanisms underlying the inflammasome activation by ER stress still remain incompletely understood.

The serine-threonine kinase receptor-interacting protein 1 (RIP1), which belongs to the RIP family, serves as a key regulator of cell survival and death in response to different cellular stress. At the crossroad of cell fate, the post-translational modification of RIP1 determines whether the cell survives or undergoes apoptosis or necrosis^10,11^. In recent several years, the mechanisms of RIP1–RIP3-mediated necrosis have gained intensive investigations and obtained big progress. In addition to its critical role in regulating cell life and death, RIP1 has also been proposed to play a role in inflammation, especially inflammasome activation induced by *Yersinia* bacteria or RNA virus infection^12–14^. Although the close connection between inflammation and cell death regulators has been more and more appreciated, the underlying mechanisms largely remain elusive. Several studies have reported that RIP1 is involved in ER stress-induced cell death^15,16^, suggesting that ER stress can signal through RIP1. To date, there is no evidence reporting whether RIP1 also contributes to inflammasome activation in physiological or pathological conditions other than microbial infection, especially in unsolved ER stress condition. In this study, we for the first time found that RIP1 contributes to the inflammasome activation induced by ER stress, through mediating mitochondrial DRP1 and production of reactive oxygen species (ROS).

## Results

### RIP1 contributes to ER stress-induced inflammasome activation

**Fig. 1:**
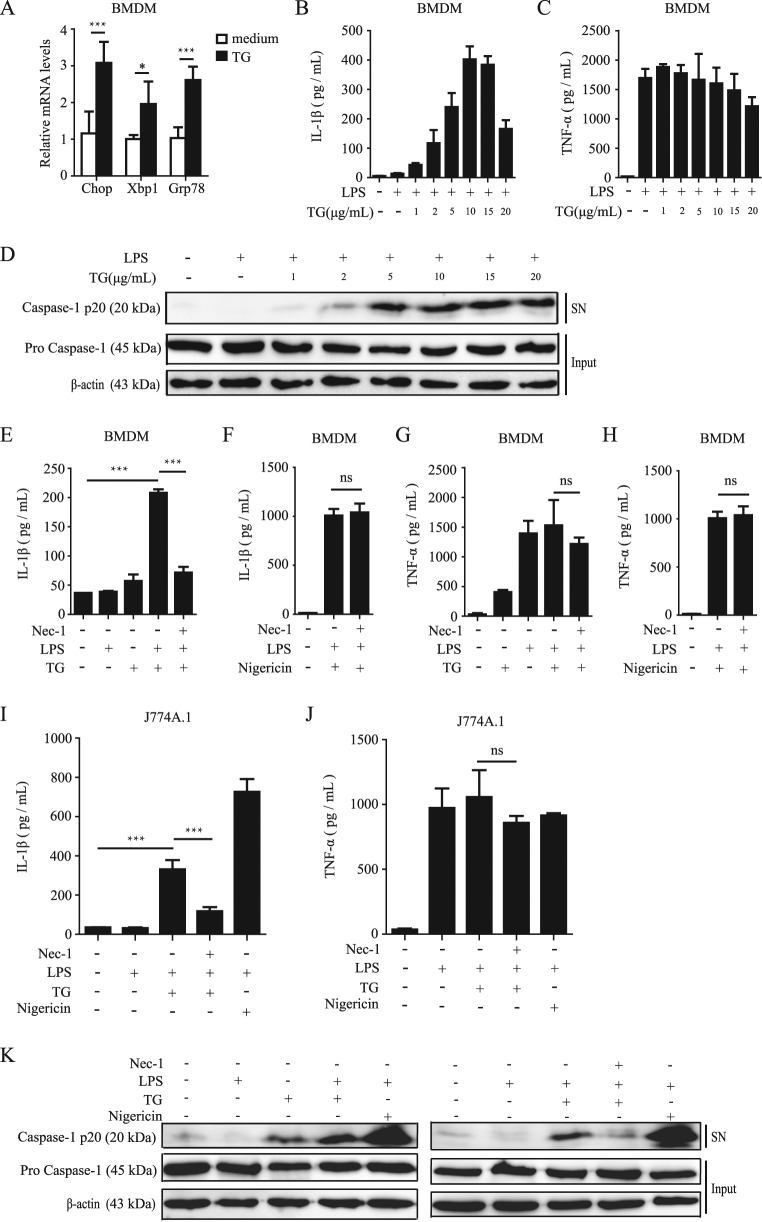
RIP1 inhibition reduces the inflammasome activation induced by ER stress. **a** The mRNA transcription level of the marker genes of ER stress, Chop, Xbp1, and Grp78, induced by TG treatment for 1 h in BMDMs. **b**, **c** BMDMs were treated with different doses of TG as indicated. The cytokine of IL-1β and TNF-α in supernatant were detected by ELISA. **d** BMDMs were stimulated as **b** and **c**, and the cleaved caspase-1(p20) in cell supernatants (SN) or caspase-1 (pro Caspase-1) and β-actin in cell lysates (Input) were detected by immunoblot. **e**– j With or without Nec-1 (20 μM) pretreatment, BMDMs or J774A.1 macrophages were treated as indicated respectively, and the cytokine release was measured by ELISA. **k** Immunoblot analysis of cleaved caspase-1 (p20) in cell supernatants (SN) or caspase-1 (pro Caspase-1) and β-actin in cell lysates (Input) of BMDMs with or without Nec-1 pretreatment (20 μM) and stimulated as indicated. Figures are representative of at least three experiments. Bars indicate means plus SD. ns not significant, **P < 0.01, *P < 0.05 (unpaired Student’s *t* test)

**Fig. 2:**
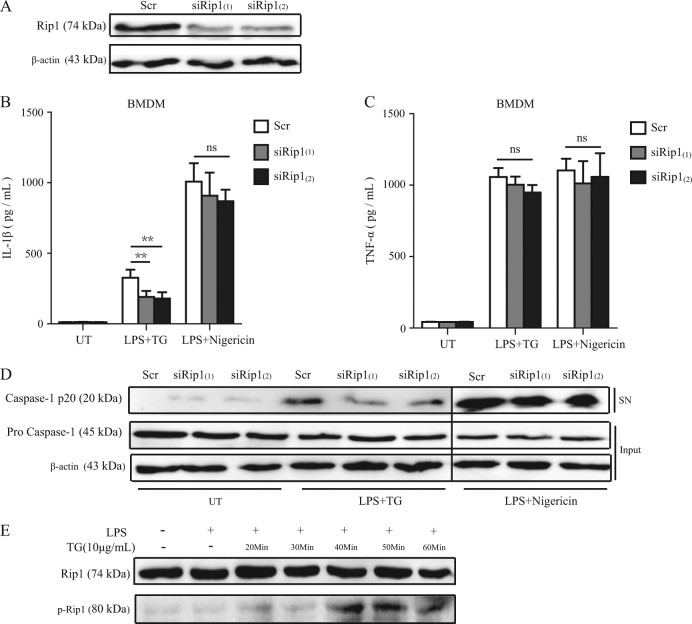
ER stress-induced inflammasome activation is severely reduced by RIP1 siRNA silencing. **a** The expression level of RIP1 in BMDMs transfected with control siRNA with a scrambled sequence (Scr) or RIP1-specific siRNA (two constructs, siRip1(1) or siRip1(2)). **b**, **c** Release of IL-1β and TNF-α by BMDMs which were first transfected with control siRNA (Scr) or RIP1-specific siRNAs and then treated with LPS plus TG or LPS plus Nigericin. UT unstimulated. **d** Immunoblot analysis of cleaved caspase-1 (p20) in cell supernatants (SN) and caspase-1 (pro-casp1) and β-actin in cell lysates (Input) of BMDMs which were first transfected with control siRNA (Scr) or RIP1-specific siRNAs and then treated with LPS plus TG or LPS plus Nigericin. **e** WT BMDMs were primed with LPS first and then treated with TG for the indicated time. The phosphorylation of RIP1 was determined by western blot with anti-phospho-RIP1 (Ser166) antibody. Figures are representative of at least three independent experiments. Bars indicate means plus SD. ns not significant, **P < 0.01, *P < 0.05 (unpaired Student’s *t* test)

To investigate the mechanism of ER stress-induced inflammasome activation, we first primed BMDMs with LPS for 3 h, followed by stimulation with ER stress-inducing drugs thapsigargin (TG). First, ER stress induced by TG was confirmed by detecting the transcription induction of ER stress markers Chop, Xbp1, and Grp78 (Fig. 1a). Next, we examined whether ER stress could induce inflammasome activation. As shown in Fig. 1b–d, LPS plus TG treatment induced obvious IL-1β production and caspase-1 cleavage. TG stimulated the secretion of IL-1β and caspase-1 cleavage in LPS-primed macrophages in a dose-dependent manner (Fig. 1b–d) and the release of IL-1β reached maximal when TG concentration was 10 μg/mL. Therefore, we treated macrophages with 10 μg/mL TG in all the following experiments unless otherwise specified. As Fig. 1e & k shown, although TG treatment without LPS priming failed to induce IL-1β secretion, it was able to induce caspase-1 cleavage in BMDMs. LPS priming further strengthened the cleavage of caspase-1 induced by TG, suggesting that ER stress triggered by TG could induce inflammasome activation by itself, but LPS is required to provide the “signal 1” to synthesize pro-IL-1β. To explore the role of RIP1 in ER stress-induced inflammasome activation, we treated cells with RIP1 kinase inhibitor Necstatin-1 (Nec-1) and found that Nec-1 pretreatment significantly decreased the IL-1β secretion induced by LPS plus TG, but not affecting the IL-1β release induced by Nigericin in both BMDMs and J774A.1 macrophages (Fig. 1e–j), which was consistent with our and others’ previous studies that Nec-1 fails to affect Nigericin-induced NLRP3 inflammasome activation^12,14^. Nec-1 also obviously suppressed the caspase-1 cleavage induced by LPS plus TG (Fig. 1k), suggesting that RIP1 kinase inhibition blocked the ER stress-induced inflammasome activation. To further confirm the role of RIP1 in this model, we knocked down expression of *Rip1* mRNA with small interfering RNA (siRNA) in BMDMs (Fig. 2a). ER stress-induced IL-1β release and caspase-1 cleavage were reduced by two different RIP1 siRNA constructs but not by control siRNA (Scr) (Fig. 2b–d). Furthermore, the production of cytokine TNF-α which is mediated by NF-κB pathway was not affected by Nec-1 treatment or RIP1 siRNAs (Figs. 1g & j and 2c), suggesting that RIP1 is not involved in the “signal 1” pathway during inflammasome activation process. Knocking down the expression of RIP1 by siRNA in J774A.1 macrophages phenocopied that in BMDMs (Supplementary Figure S1), confirming the role of RIP1 in ER stress-induced inflammasome activation in different cell types.

Since our results suggested that RIP1 is required in ER stress-induced inflammasome activation, we wandered whether ER stress could activate RIP1 or not. As Fig. 2e shown, TG started to induce the phosphorylation of RIP1 (Ser166) 20 min after administration and the phosphorylation of RIP1 turned to be stronger at 40 and 50 min, suggesting that TG was able to activate RIP1 at an early time point. Taken together, these results indicated that RIP1 was activated by ER stress and was required in ER stress-induced inflammasome activation.

### RIP3 deficiency does not influence the inflammasome activation induced by ER stress

**Fig. 3:**
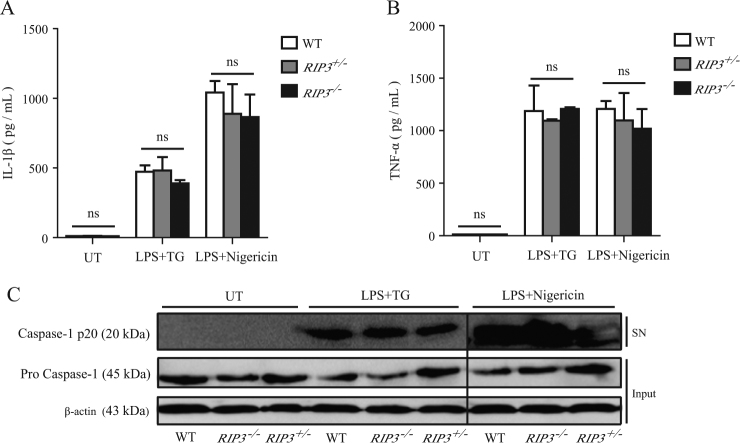
ER stress-induced inflammasome activation does not depend on RIP3. **a**, **b** Secretion of IL-1β and TNF-α by WT, *RIP3*^+/−^, and *RIP3*^−/−^ BMDMs stimulated with LPS plus TG or LPS plus Nigericin. UT unstimulated. **c** Immunoblot analysis of cleaved caspase-1 (p20) in cell supernatants (SN) and caspase-1 (pro-casp1) and β-actin in cell lysates (Input) of WT, *RIP3*^+/−^, and *RIP3*^−/−^ BMDMs stimulated with LPS plus TG or LPS plus Nigericin. Figures are representative of at least three independent experiments. Bars indicate means plus SD. ns not significant (unpaired Student’s *t* test)

RIP1 cooperates with RIP3, another RIP family member, to mediate the occurrence of necropotosis^11,17,18^. To address whether RIP3 is also required for ER stress-mediated inflammasome activation, we treated BMDMs from RIP3-deficient mice^19^ with LPS and TG. IL-1β secretion was comparable in WT, *Rip3*^−/−^, and *Rip3*^+/−^ BMDMs (Fig. 3a). Similarly, caspase-1 cleavage induced by LPS plus TG was not reduced in *Rip3*^−/−^ BMDMs (Fig. 3c). These results suggested that RIP3 deficiency did not affect the inflammasome activation induced by ER stress and this result was in agreement with published report^6^.

### DRP1 is required for RIP1-mediated inflammasome activation induced by ER stress

**Fig. 4:**
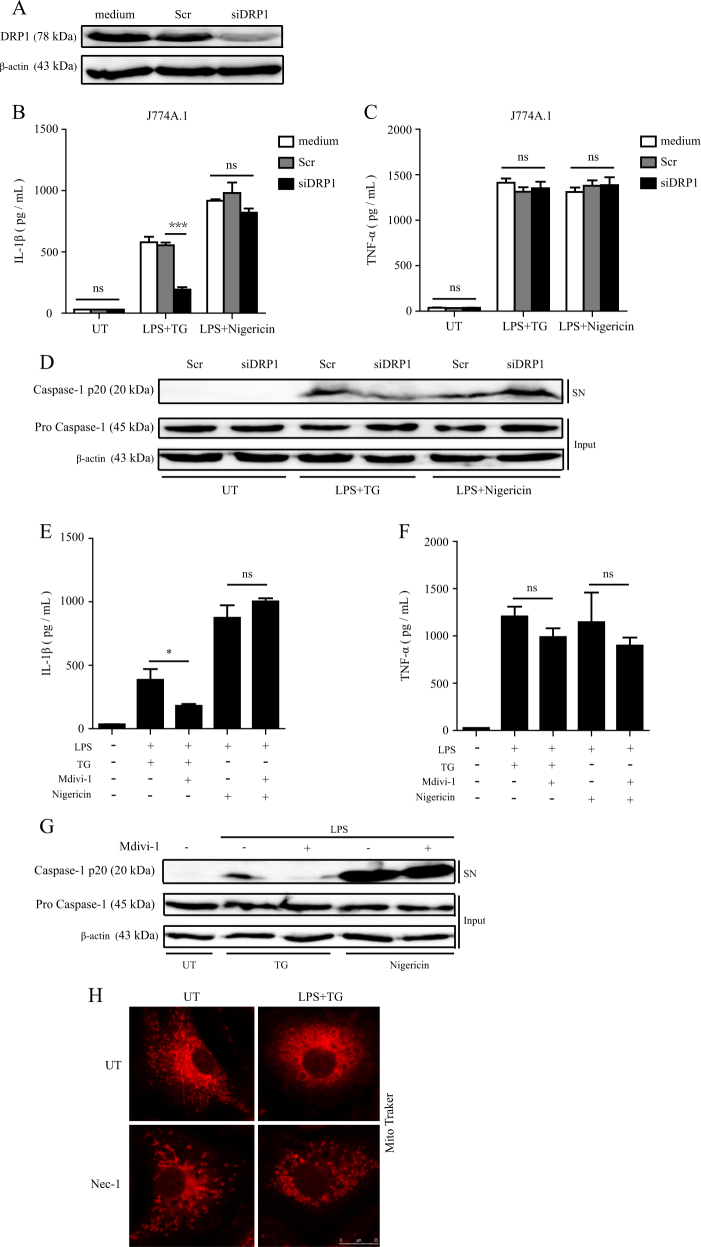
DRP1 contributes to ER stress-induced inflammasome activation. **a** The expression level of DRP1 in J774A.1 macrophages transfected with control siRNA with a scrambled sequence (Scr) or DRP1-specific siRNA. **b**, **c** Release of IL-1β and TNF-α by J774A.1 macrophages which were first transfected with control siRNA (Scr) or DRP1-specific siRNA and then treated with LPS plus TG or LPS plus Nigericin. **d** Immunoblot analysis of cleaved caspase-1 (p20) in cell supernatants (SN) and caspase-1 (pro-casp1) and β-actin in cell lysates (Input) of J774A.1 macrophages which were first transfected with control siRNA (Scr) or DRP1-specific siRNA and then treated with LPS plus TG or LPS plus Nigericin. **e**, **f** Secretion of IL-1β and TNF-α by BMDMs stimulated by LPS plus TG or LPS plus Nigericin with or without DRP1 inhibitor Mdivi-1 pretreatment. **g** Immunoblot of cleaved caspase-1 (p20) in cell supernatants (SN) and caspase-1 (pro-casp1) and β-actin in cell lysates (Input) of BMDMs stimulated with LPS plus TG or LPS plus Nigericin with or without Mdivi-1 pretreatment. UT, unstimulated. **h** Confocal microscopy analysis of BMDMs with or without Nec-1 pretreatment and then stimulated with LPS plus TG. Cells were stained with MitoTracker. Scale bar, 25 μm. Figures are representative of at least three independent experiments. Bars indicate means plus SD. ns not significant, **P < 0.01, *P < 0.05 (unpaired Student’s *t* test)

RIP1 has been reported to mediate RNA virus-induced inflammasome activation through activating the mitochondria fission protein DRP1 to drive mitochondrial damage^13^. To investigate the downstream mechanism of RIP1-mediated inflammasome activation induced by ER stress, we first examined the role of DRP1. As Fig. 4b, d shown, when *Drp1* expression was silenced by siRNA in J774 A.1 macrophages (Fig. 4a), LPS plus TG-induced IL-1β secretion and caspase-1 cleavage was obviously impaired, while LPS plus TG-induced TNF-α production was not affected (Fig. 4c). We confirmed the effect of DRP1 siRNAs in BMDMs (Supplementary Figure S2). We also used Mdivi-1, a selective inhibitor of DRP1, to confirm the role of DRP1. The results indicated that Mdivi-1 pretreatment significantly inhibited IL-1β production as well as caspase-1 cleavage induced by ER stress in BMDMs (Fig. 4e–g), supporting the results of DRP1 siRNA which suggested a critical role of DRP1 in ER stress-induced inflammasome activation. Because DRP1 mediates mitochondria fission^20^, which has been reported to be involved in RNA virus-induced NLRP3 inflammasome activation^13^, we therefore used confocal microscopy to detect the mitochondrial fission. As Fig. 4h shown, LPS plus TG promoted mitochondrial fission in WT BMDMs and the RIP1 inhibitor Nec-1 obviously reduced the occurrence of fission, suggesting that DRP1 might be regulated by RIP1. Taken together, these results suggested that DRP1 plays an important role in ER stress-induced inflammasome activation and may localize downstream of RIP1.

### ROS is involved in ER stress-induced inflammasome activation

**Fig. 5:**
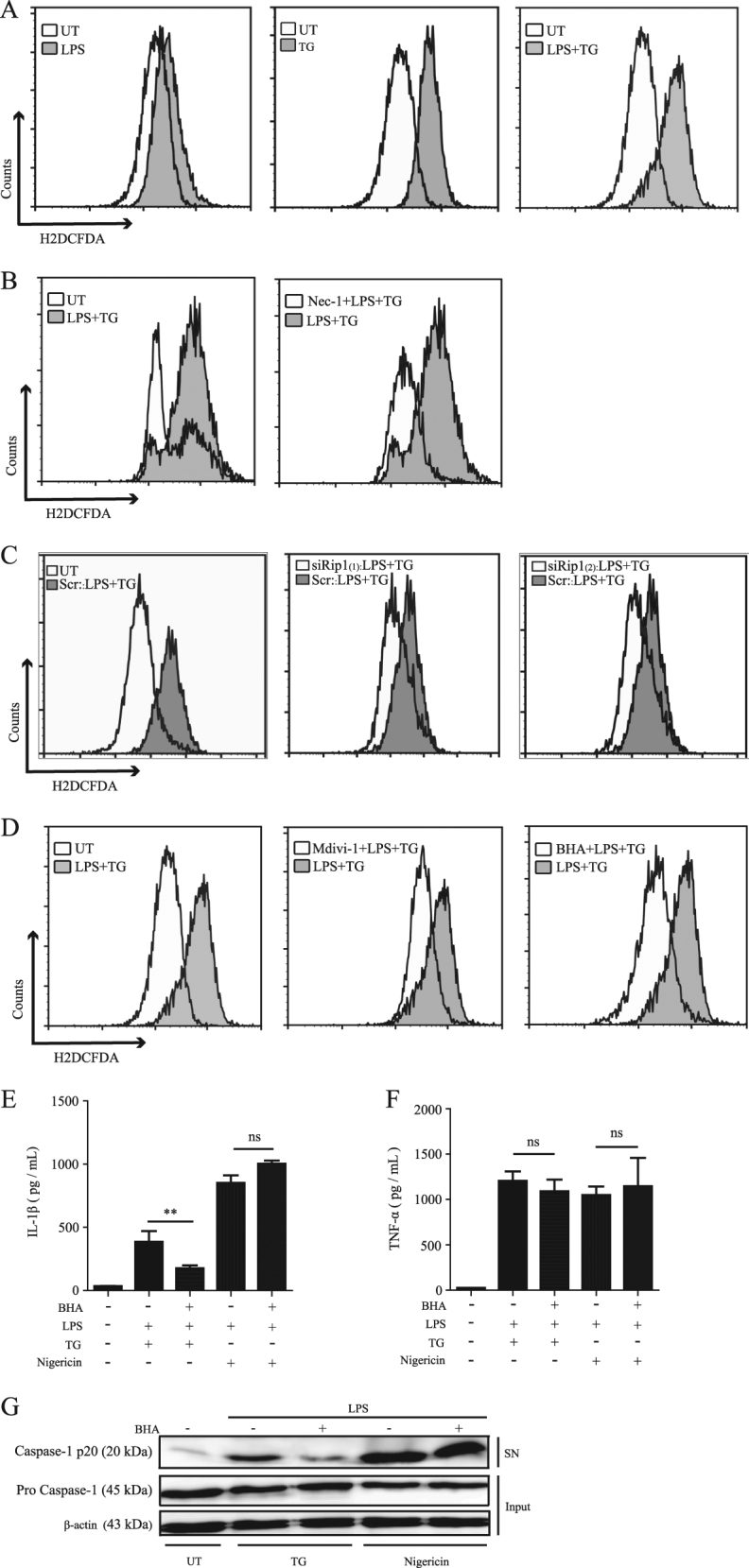
ROS is involved in inflammasome activation induced by ER stress. **a** ROS production detected by flow cytometry in LPS, TG, or LPS plus TG-treated BMDMs (UT unstimulated). **b** ROS production in LPS plus TG-treated BMDMs with or without Nec-1 pretreatment. **c** ROS production in LPS plus TG-treated BMDMs transfected with control siRNA (Scr) or RIP1-specific siRNAs. **d** ROS production in LPS plus TG-treated BMDMs with or without Midivi-1 or BHA pretreatment. **e**, **f** Secretion of IL-1β and TNF-α by BMDMs stimulated by LPS plus TG or LPS plus Nigericin with or without antioxidant BHA pretreatment. **g** Immunoblot of cleaved caspase-1 (p20) in cell supernatants (SN) and caspase-1 (pro caspase-1) and β-actin in cell lysates (Input) of BMDMs stimulated with LPS plus TG or LPS plus Nigericin with or without BHA pretreatment. Figures are representative of at least three independent experiments. Bars indicate means plus SD. ns not significant, **P < 0.01, *P < 0.05 (unpaired Student’s *t* test)

DRP1-mediated mitochondrial fission can lead to ROS production^20^. ROS has been proposed to mediate inflammasome activation in different models^21–23^. Therefore, we asked whether ROS is involved in ER stress-induced inflammasome activation. To answer this question, first we detected whether ER stress here induces ROS generation or not. Using flow cytometer with fluorescence probe H_2_DCFDA, the results indicated that TG or LPS plus TG treatment both prompted obvious ROS production (Fig. 5a), which was inhibited by RIP1 inhibitor Nec-1 pretreatment, or RIP1 siRNA transfection, or DRP1 inhibitor Mdivi-1 pretreatment (Fig. 5b–d). These results implicated that both RIP1 and DRP1 might mediate ROS induction under ER stress stimulus. Next we used ROS inhibitor BHA to treat cells before TG stimulation and as the results shown, BHA did obviously reduce the production of ROS induced by LPS plus TG stimulation (Fig. 5d). BHA also significantly decreased the secretion of IL-1β and caspase-1 cleavage induced by LPS plus TG (Fig. 5e–g), without influencing the production of TNF-α (Fig. 5f). Together, these results suggested ROS production was involved in ER stress-induced inflammasome activation and its production was mediated by RIP1 and DRP1.

### Cell death is not involved

**Fig. 6:**
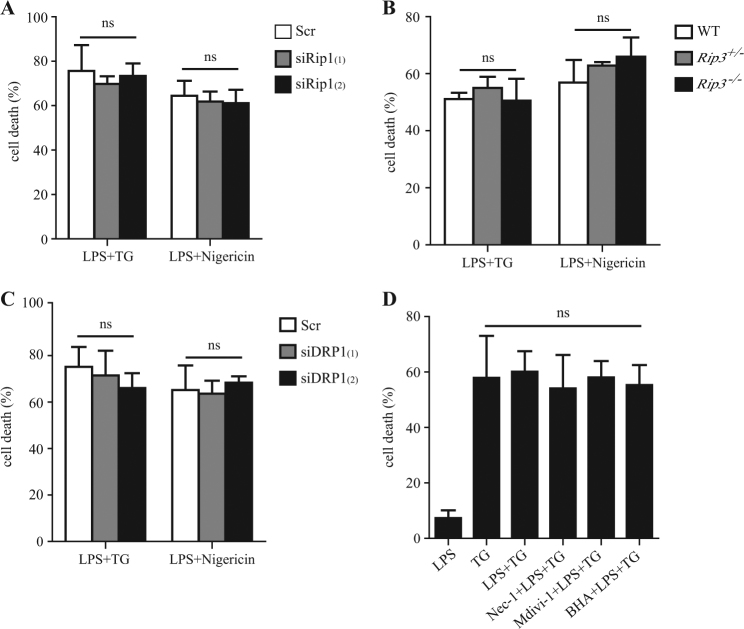
Cell death is not involved in ER stress-induced inflammasome activation. **a**, **c** Cell death induced by ER stress inducer TG in BMDMs transfected with control siRNA (Scr) or RIP1-specific siRNAs (siRip1) or DRP1-specific siRNAs (siDRP1). The cell death rate was measured by LDH release assay. **b** Cell death induced by LPS plus TG treatment or LPS plus Nigericin treatment in WT, *RIP3*^*+/*^^−^, and *RIP3*^−/−^ BMDMs. **d** Cell death induced by LPS plus TG with or without pretreatment of various inhibitors as indicated. Results are from three independent experiments with biological duplicates in each. Bars indicate means plus SD. ns not significant (unpaired Student’s *t* test)

We also investigated whether cell death was involved in ER stress-induced inflammasome activation or not, since RIP1 is a key regulator of cell survival and death in response to different cellular stress. To examine whether the impaired IL-1β release and caspase-1 cleavage by RIP1 inhibition or silencing might be due to less cell death, we detected the cell death induced by ER stress with RIP1 inhibitor or RIP1 siRNAs treatment. As shown in Fig. 6a, c, d, the cytotoxicity induced by LPS plus TG was not altered by RIP1 siRNA or DRP1 siRNA transfection, or by Nec-1, Mdivi-1, or BHA pretreatment, suggesting that the cell death induced by ER stress was not mediated by RIP1, DRP1 or ROS. Consistently, the cytotoxicity was similar between *Rip3*^−/−^, WT, and *Rip3*^+/−^BMDMs (Fig. 6b), confirming that RIP1–RIP3-mediated necroptosis did not account for TG-induced cell death. Therefore, the RIP1-mediated inflammasome activation induced by ER stress was not attributable to RIP1-regulated cell death.

### RIP1 and DRP1 are also required for inflammasome activation induced by another ER stress inducer

**Fig. 7:**
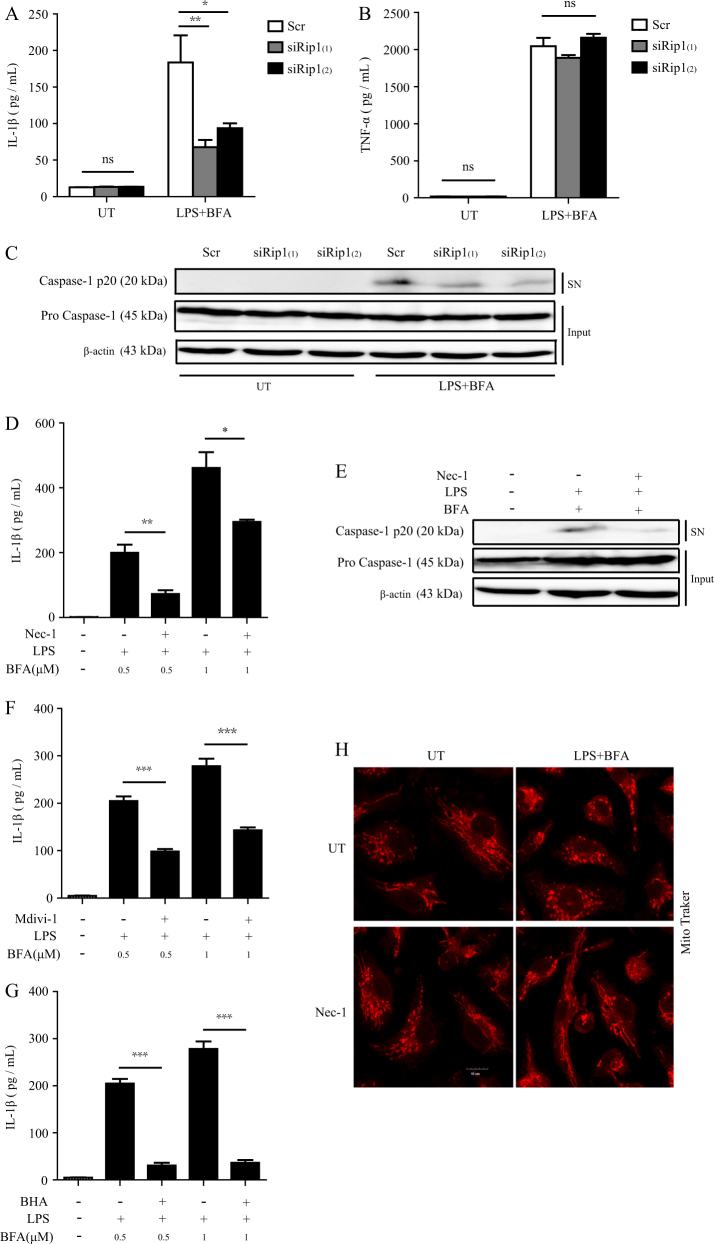
RIP1 and DRP1 are also required for inflammasome activation induced by another ER stress inducer BFA. **a**, **b** Release of IL-1β and TNF-α by BMDMs which were first transfected with control siRNA (Scr) or RIP1-specific siRNAs and then treated with LPS plus BFA (1 μM) for 12 h. UT unstimulated. **c** Immunoblot analysis of cleaved caspase-1 (p20) in cell supernatants (SN) and caspase-1 (pro-casp1) and β-actin in cell lysates (Input) of BMDMs which were treated as **a** and **b**. **d**–**g** Secretion of IL-1β (**d**, **f**, **g**) or immunoblot analysis of cleaved caspase-1 (p20) in cell supernatants (SN) (**e**) of BMDMs which were stimulated by LPS plus BFA (1 μM or as indicated) for 12 h. **h** Confocal microscopy analysis of BMDMs with or without Nec-1 pretreatment and then stimulated with LPS plus BFA (1 μM). Cells were stained with MitoTracker. Scale bar, 25 μm. Figures are representative of at least three independent experiments. Bars indicate means plus SD. ns not significant. ***P < 0.001, **P < 0.01, *P < 0.05 (unpaired Student’s *t* test)

To further confirm the role of RIP1 and DRP1 in ER stress-induced inflammasome activation, we used another classical ER stress-inducing drug, Brefeldin A (BFA), which is an ER–Golgi transport inhibitor^24,25^, to treat macrophages. Consistent with the results by using TG, BFA-induced IL-1β release, and caspase-1 cleavage were significantly reduced by RIP1 siRNAs transfection compared to control siRNA (Scr) (Fig. 7a–c). Similarly, RIP1 knockdown did not affect the secretion of TNF-α induced by LPS plus BFA (Fig. 7b), suggesting that knocking down the expression of RIP1 only influenced the “signal 2” and did not affect “signal 1” during BFA-induced inflammasome activation. Moreover, BFA-induced IL-1β production and caspase-1 activation were also inhibited by pretreatment with the RIP1 inhibitor Nec-1 (Fig. 7d, e). These results suggest that the role of RIP1 in ER stress-induced inflammasome activation is not limited to TG stimulation, but is involved in different ER stress models.

We also examined the role of DRP1 and ROS in BFA-induced inflammasome activation. In agreement with TG treatment, BFA-induced IL-1β release was markedly inhibited by pretreatment with DRP1 inhibitor Mdivi-1 or antioxidant BHA respectively (Fig. 7f, g). Moreover, LPS plus BFA induced obvious mitochondrial fission in WT BMDMs and Nec-1 efficiently suppressed the occurrence of mitochondrial fission (Fig. 7h). These results together suggested that BFA-induced inflammasome activation shared the same mechanisms as TG, and RIP1–DRP1–ROS pathway played a critical role in it.

## Discussion

ER stress has been reported to induce NLRP3 inflammasome activation in different cell types^5–8^. However, the underlying mechanism is not fully understood. In this study, we identified RIP1 as a critical regulator in ER stress-induced inflammasome activation in macrophages. RIP1 inhibition or silencing severely impaired the inflammasome activation, resulting in reduced caspase-1 cleavage and IL-1β secretion induced by two different ER stress inducers, TG and BFA. However, the close partner of RIP1 in mediating necroptosis, RIP3, was not required in such process, as RIP3 deficiency did not affect the inflammasome activation induced by ER stress. We also demonstrated that ER stress can induce RIP1 phosphorylation at Ser166, DRP1 and ROS functioned as the effectors downstream of RIP1 to mediate inflammasome activation. Our results suggest that RIP1 plays an important role in inflammatory signaling pathways under ER stress conditions.

RIP1 is well known to act as a key regulator at the crossroad between cell survival and death downstream of different receptors^10,11^. Recent studies reported that in addition to its critical role in determining life or death, RIP1 is also involved in mediating inflammasome activation induced by *Yersinia* bacteria or RNA virus infection^12–14^. Gaidt et al. reported that RIP1 mediates an “alternative inflammasome” activation triggered by LPS-TLR4-TRIF signaling upstream of NLRP3 in human monocytes^26^. Here we have broadened the current knowledge that RIP1 also contributes to ER stress-induced inflammasome activation, suggesting that RIP1-mediated inflammasome activation might be an alternative supplement to classical inflammasome signaling. Regarding the mechanism underlying RIP1-mediated inflammasome activation, it seems to be cell type-dependent and cellular context-dependent.

RIP1 acts in synergy with RIP3 to induce necroptosis. Regarding the role of RIP3 in inflammasome activation, it remains controversial. In our study, in contrast to RIP1, RIP3 is dispensable for ER stress-induced inflammasome activation, which is consistent with the study by Shenderov et al. who found that caspase-8 and TRIF, but not RIP3, are required for ER stress-induced caspase-1 cleavage and IL-1β secretion^6^. Our previous study also demonstrated that inflammasome activation induced by Gram-negative bacteria *Yersinia pestis* infection was abrogated in *Rip1*^−/−^ and *Rip3*^−/−^*Caspase-8*^−/−^ macrophages, but not in *Rip3*^−/−^ cells^14^, which is consistent with another independent study using *Yersinia pseudotuberculosis*^12^. However, Wang et al. reported that both RIP1 and RIP3 are required for inflammasome activation induced by RNA virus, including vesicular stomatitis virus (VSV), Sendai virus and influenza virus^13^, although Moriwaki and Chan stated in a review that they were not able to detect RIP3-dependent IL-1β secretion in response to VSV infection (their unpublished observation)^27^. In addition, Kang et al. reported that RIP3-mediated activation of the NLRP3 inflammasome is blocked by caspase-8 and caspase-8 deficiency facilitates inflammasome activation by LPS treatment in dendritic cells, which can be inhibited by RIP3 deletion^28^. These studies suggest that in inflammasome signaling, RIP3 and caspase-8 are highly intertwined and the role of RIP3 in inflammasome activation might rely on the status of caspase-8 and is cell type-dependent and cellular context-dependent.

Our results revealed that DRP1 acts downstream of RIP1 to mediate ER stress-induced inflammasome activation, as Nec-1 inhibited the DRP1-mediated mitochondrial fission induced by LPS plus TG. Moreover, both DRP1 inhibition by Mdivi-1 and DRP1 silencing by specific siRNAs decreased the IL-1β production and caspase-1 cleavage triggered by ER stress. The role of DRP1 here is in agreement with the study by Wang et al., who have also indicated that RIP1 interacts with and phosphorylates DRP1 to promote DRP1 activation upon RNA virus infection^13^. DRP1 is a GTPase which functions as a main regulator in mitochondrial fission. It normally localizes in cytosol and when cell encounters stress signals, DRP1 is phosphorylated at Ser616 and translocalizes to mitochondrial outer membrane to mediate mitochondrial fission^29^. DRP1 has been reported to contribute to inflammasome activation through inducing ROS generation during mitochondrial fission^13,30^. Moreover, Wang et al. have also indicated that RIP1 interacts with DRP1^13^. The study by Dara et al. demonstrated that RIP1 knockdown abrogated the translocation of DRP1 to mitochondria^31^. Taken together with previous reports and our results, RIP1–DRP1–ROS might serve as another mechanism to mediate inflammasome activation under different conditions, yet the detailed underlying mechanism still demands further clarification.

It has been established that ER stress can induce inflammasome activation and mature IL-1β secretion either by using chemical ER stress inducers or under hyperglycemia or hyperlipidemia conditions^6,32–35^. The underlying mechanism has gained lots of investigations. Several studies reported that one of the UPR sensors, IRE1α, is responsible for mediating inflammasome activation induced by ER stress^32–35^. We have obtained similar results that IRE1α inhibitor, 4u8c, significantly inhibited the caspase-1 cleavage and IL-1β secretion induced by LPS plus TG (data not shown). However, about the mechanism downstream of IRE1α, it remains controversial. Both Oslowski et al. and Lerner et al. published that IRE1α prompts NLRP3 inflammasome activation and cell apoptosis through inducing thioredoxin-interacting protein (TXNIP) in islet β cells or human THP-1 cells^32,33^, although neither of them directly detected IL-1β secretion or caspase-1 cleavage in TXNIP-deficient cells. In contrast, Tufanli et al. reported that lipotoxic ER stress-induced inflammasome activation in macrophages is independent of TXNIP induction^35^, suggesting that the role of TNXIP is still conflicting and needs more exploration by utilizing TXNIP knockout cells.We have examined the effect of IRE1α inhibitor, 4u8c, on the phosphorylation of RIP1 and the results indicated that 4u8c pretreatment did not affect the phosporylation of RIP1 induced by ER stress inducers, including both TG and BFA (data not shown). Since RIP1 inhibition or knockdown did not completely block the secretion of IL-1β induced by ER stress, we hypothesized that there might be different alternative mechanisms regulating the inflammasome activation in ER stress condition. RIP1 and IRE1α might be involved in different mechanic pathways. The relationship between UPR sensors and RIP1 needs future investigations.

ER stress is associated with various diseases, including obesity, diabetes, atherosclerosis, neurodegenerative disorders, and cancer^2,36,37^. Accumulative evidence suggest that ER stress contributes to the induction of chronic inflammation, possibly via inducing inflammasome activation and thus cytokine IL-1β generation or causing cell death which leading to inflammatory molecule release^4,5^. The interaction between ER stress and inflammation might play a critical role in disease occurrence and progression. In the present study, we identified RIP1 as an important mediator in ER stress-induced inflammasome activation, through regulating DRP1 and ROS generation. Several studies have previously demonstrated that RIP1 is also involved in ER stress-induced cell death^15,16^, although our results indicated that cell death fails to account for RIP1-mediated inflammasome activation by ER stress. However, these findings suggest that RIP1 might be linked to ER stress-associated inflammation through regulating both inflammasome activation and cell death, thus be capable to serve as a promising therapeutic target for treating diseases caused by unresolved ER stress.

## Materials and methods

### Regents

Necrostatin-1 (Nec-1) was purchased from EnzoLifesciences. Anti-Caspase-1 (p20) (mouse) mAb (Casper-1) was from Adipogen. Anti-RIP1 (mouse) mAb was from BD Transduction. Phospho-RIP (Ser166) Rabbit mAb was from Cell Signaling Technology. Thapsigargin (TG), Brefeldin A (BFA), Butyl hydroxyl anisd (BHA), and Lipopolysaccharides (LPS) was from Sigma-Aldrich. Murine M-CSF was from Peprotech. Nigericin was from Merck-Millipore and Mdivi-1 was from Selleckchem. Mitotracker® Red CMXRos was from YEASEN.

### Cell culture and stimulation

Mouse bone marrow-derived macrophages (BMDMs) were derived by maturing bone marrow cells for 6–7 days in the presence of 10 ng/mL M-CSF as previously described^38^. J774A.1 macrophages, which were purchased from the Cell Bank of the Chinese Academy of Sciences (Shanghai, China), were cultured in DMEM (GIBCO) with 10% heat-inactivated FBS and 1% penicillin-streptomycin. To induce ER stress, cells were primed with LPS (100 ng/mL) for 3 h and then stimulated with TG (10 μg/mL) for 4 h or BFA (1 μM) for 12 h. In some experiments, BMDMs or J774A.1 macrophages were pre-treated with RIP1 inhibitor Nec-1 (20 μM or 40 μM) or Mdivi-1(10 μM) or BHA (50 μM) for 1 h before TG or BFA treatment. Supernatants were collected for immunoblot assay, cytokine analysis, and LDH assay. Cell lysate was harvested for immunoblot analysis.

### Mice

C57Bl/6 mice were purchased from Model Animal Research Center of Nanjing University (Nanjing, China). *Rip3*^+/−^ mice on a C57Bl/6 background, which gave birth to *Rip3*^+/+^ (WT), *Rip3*^+/−^, and *Rip3*^−/−^ littermates, were kindly provided by Dr. Jiahuai Han (Xiamen University, China). All animals were maintained under standard laboratory conditions, with access to food and water *ad libitum*. All animal experiments were performed under protocols approved by the Animal Care and Use Committee at Nanjing University of Science & Technology.

### Immunoblot assay

Cells were lysed in cell lysis buffer (Beyotime) with protease inhibitor (Beyotime) and phosphatase inhibitor (Beyotime). Proteins were separated by SDS-PAGE(10–15% gel), transferred to 0.22 μM PVDF membranes (Biosharp), blocked with 5% skim milk powder (Biosharp) in TBS-T (0.1% TWEEN-20). Then membranes were incubated with primary antibodies overnight, washed with TBS-T and incubated with secondary antibodies at room temperature for 1 h. Chemiluminescent Substrate System from KPL was utilized for final detection.

### siRNA-mediated gene silencing in cells

BMDMs or J774A.1 macrophages were plated in 6-well or 96-well plates and then were transfected with Rip1 or DRP1 siRNAs or their negative siRNA control (Scr) (synthesized by GenePharma) using LipofectamineRNAiMAX (Invitrogen) according to the manufacturer’s instructions. 48 h after transfection, LPS-primed BMDMs were stimulated with TG (10 μg/mL) for 4 h, or BFA (1 μM) for 12 h or Nigericin (5 μM) for 1 h. Supernatants were collected for immunoblot assay, cytokine analysis or LDH assay. Cell lysates were harvested for immunoblot assay. The siRNA sequences are as follows (5′–3′)^13^:

siRip1_(1)_:GCCAAAUCUAAGCCAAAUGUATT; siRip1_(2)_:CGUGACUUUCACAUUAAGAUATT; siDRP1_(1)_:GGCAAUUGAGCUAGCGUAUAUTT; siDRP1_(2)_:CUAUAAUGCAUGCACUAUUUATT.

### Enzyme-linked immunosorbent assay

Cytokines (IL-1β and TNF-α) in cell supernatants were measured by ELISA according to the manufacturer’s instructions using ELISA kits from eBioscience.

### ROS measurement

BMDMs were plated in 6-well plates for 2 h before treatment. Some cells were transfected with Rip1 or DRP1 siRNAs or their negative siRNA control using LipofectamineRNAiMAX (Invitrogen) according to the manufacturer’s instructions. With or without pretreatment by different inhibitors, cells were primed with LPS (100 ng/mL) for 3 h and then stimulated with TG (10 μg/mL) for 1 h. Next fresh FBS-free medium with H_2_DCFDA (20 μM) (Aladdin) were added for another 20 min. Cells were then detached in PBS with 0.5 M EDTA, washed twice, and resuspended in PBS. ROS production was detected by NovoCyteflow cytometer (ACEA, US).

### LDH release assay

Cell death was quantified by detecting the lactate dehydrogenase (LDH) release using kit from Beyotime company following the manufacturer’s instruction. Background LDH release was determined by assaying supernatants from unstimulated cells. Total LDH was determined from unstimulated cells which had been lysed by releasing agent. The cell death rate was calculated as: (sample LDH − background LDH) / (total LDH − background LDH) × 100%.

### q-PCR analysis

BMDMs were stimulated with TG (10 μg/mL) for 1 h. Then TRIzol (Invitrogen) reagent was added to cells and total RNA was extracted according to the manufacturer’s instructions. Next cDNA was synthesized using Reverse Transcription Kit (Invitrogen). SYBY-Green Premix (Toyobo) was used by the ABI 7300 real-time PCR system. The sequences of primers for qPCR were as follows:

Grp78, 5′-CGAGGAGGAGGACAAGAAGG-3′ (forward) 5′-TCAAGAACGGGCAAGTTCCAC-3′ (reverse); Xbp1, 5′-GAGTCCGCAGCAGGTG-3′ (forward) 5′-AGGGTACCTGAGACTGTG-3′ (reverse); Chop, 5′-CTGCCTTTCACCTTGGAGAC-3′ (forward) 5′-ATAGAGTAGGGGTCCTTTGC-3′ (reverse); GAPDH, 5′-AGGTCGGTGTGAACGGATTTG-3′ (forward) 5′-TGTAGACCATGTAGTTGAGGTCA-3′ (reverse).

### Confocal microscopy

BMDMs were plated on coverslips for 8–10 h and then were primed with LPS (100 ng/mL) for 3 h and then stimulated with TG (10 μg/mL) for 1 h or BFA (1 μM) for 12 h. MitoTraker Red (200 nM) (Aladdin) were added for another 30 min. After being washed three times with PBS, the cells were fixed for 15 min with 4% PFA and were washed 3 times with PBS. The coverslips were fixed on glass slide with 50% PBS glycerin. Two-photon confocal laser scanning microscopy (Lei TCS SPB-MaiTai MP) was used for analysis. Scanning fileds were randomly selected and at least 50 cells were counted in each sample.

### Statistical analysis

All experiments were repeated at least three times. Data were analyzed with GraphPad Prism® 5.0. Unpaired Student’s *t* test was used to calculate the the two-tailed *P* values. Difference was considered significant when *P* values were below 0.05.

## Electronic supplementary material

Supplementary Figure S1

Supplementary Figure S2
