# Supplementary material for: The kinase receptor-interacting protein 1 is required for inflammasome activation induced by endoplasmic reticulum stress
Source: Cell Death Dis. 2018 May 29;9(6):641. doi: 10.1038/s41419-018-0694-7 (PMC5974395; doi:10.1038/s41419-018-0694-7)
Supplement: Supplementary file 1 — Supplementary Figure S1 [file 41419_2018_694_MOESM1_ESM.pdf]

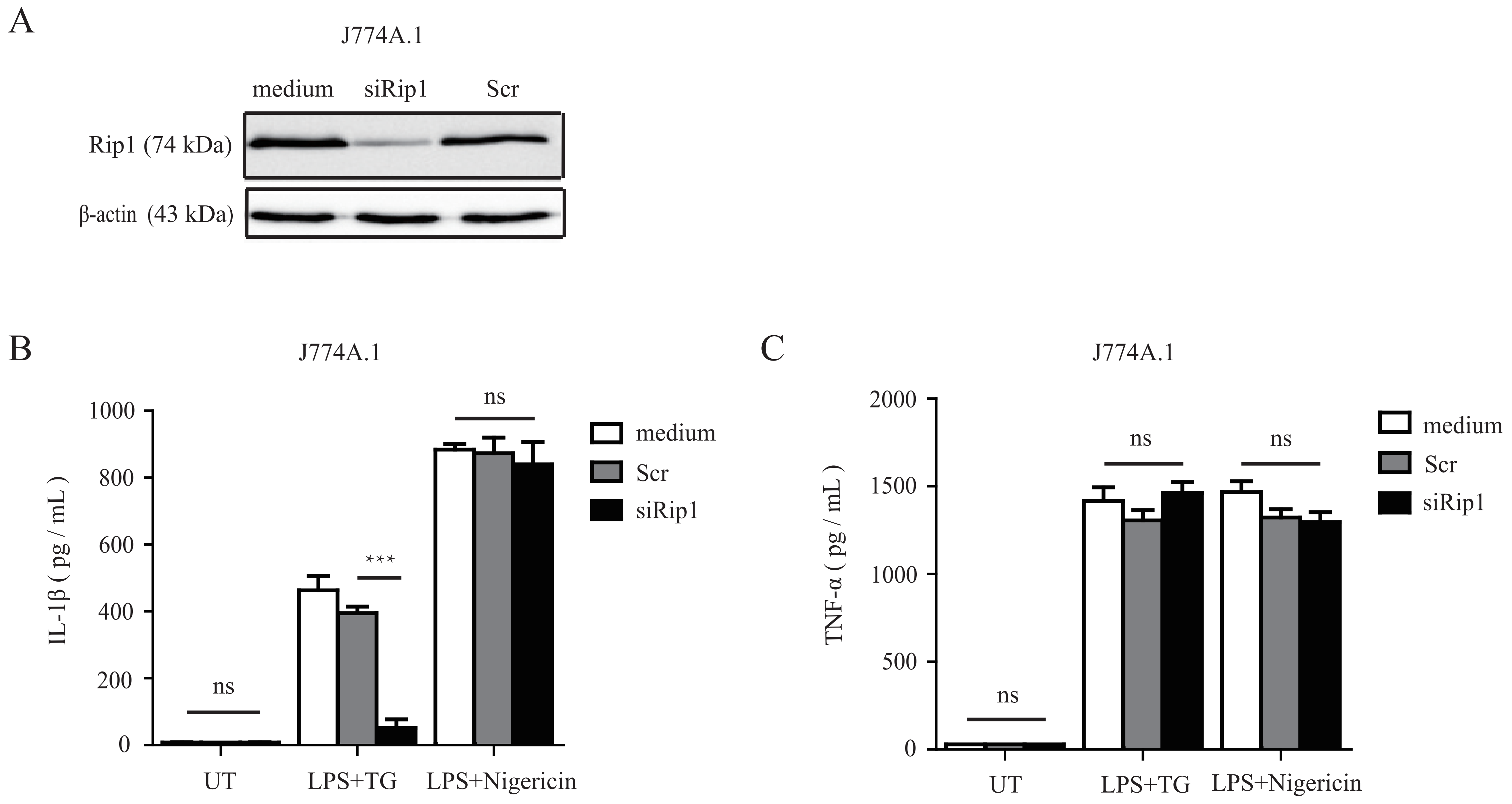

Figure S1 RIP1 siRNA reduces the inflammasome activation induced by ER stress in J774A.1 macrophages. (A) The expression level of RIP1 in BMDMs transfected with control siRNA with a scrambled sequence (Scr) or RIP1-specific siRNA. (B and C) Release of IL-1β and TNFα by BMDMs which were first transfected with control siRNA (Scr) or RIP1-specific siRNA and then treated with LPS plus TG or LPS plus Nigericin. UT, unstimulated. Figures are representative of at least three independent experiments. Bars indicate means plus SD. ns, not significant, **$P < 0.01$, *$P < 0.05$ (unpaired Student's t test).
