# Supplementary material for: The kinase receptor-interacting protein 1 is required for inflammasome activation induced by endoplasmic reticulum stress
Source: Cell Death Dis. 2018 May 29;9(6):641. doi: 10.1038/s41419-018-0694-7 (PMC5974395; doi:10.1038/s41419-018-0694-7)
Supplement: Supplementary file 2 — Supplementary Figure S2 [file 41419_2018_694_MOESM2_ESM.pdf]

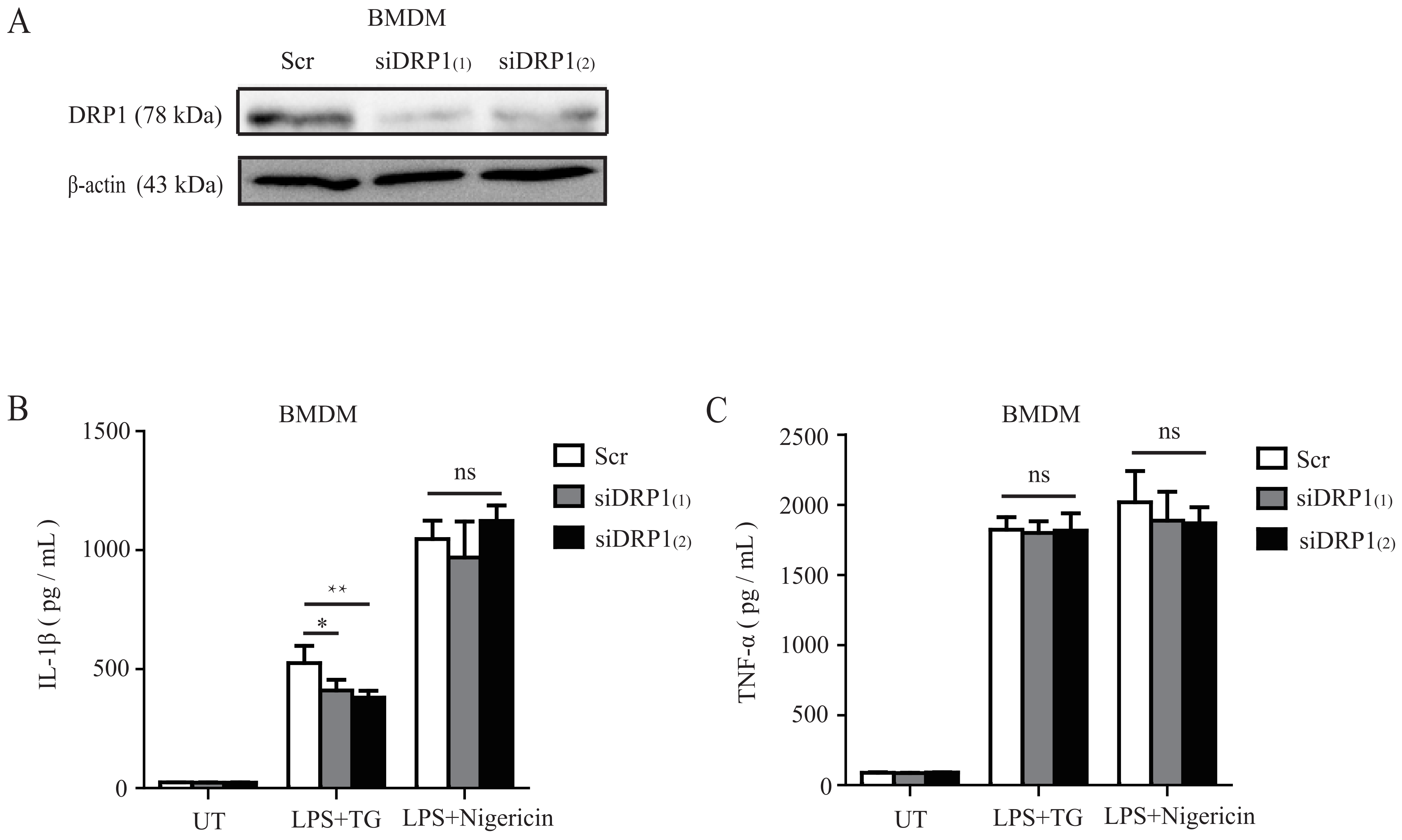

Figure S2 ER stress-induced inflammasome activation is severely reduced by DRP1 siRNA silencing in BMDMs. (A) The expression level of DRP1 in BMDMs transfected with control siRNA with a scrambled sequence (Scr) or DRP1-specific siRNA (two constructs, siDRP1(1) or siDRP1(2)). (B and C) Release of IL-1β and TNFα by BMDMs which were first transfected with control siRNA (Scr) or DRP1-specific siRNAs and then treated with LPS plus TG or LPS plus Nigericin. UT, unstimulated. Figures are representative of at least three independent experiments. Bars indicate means plus SD. ns, not significant, **$P < 0.01$, *$P < 0.05$ (unpaired Student's t test).
